# The world as witty agent—Donna Haraway on the object of knowledge

**DOI:** 10.3389/fpsyg.2024.1389575

**Published:** 2024-09-20

**Authors:** Jasmin Trächtler

**Affiliations:** Department of Humanities and Theology, Institute for Political Science and Philosophy, Technical University Dortmund, Dortmund, Germany

**Keywords:** scientific objectivity, feminist philosophy of science, Donna Haraway, epistemic agency, situated knowledges

## Abstract

In her essay “Situated Knowledges,” the biologist and philosopher of science Donna Haraway tackles the question of scientific objectivity from a feminist perspective and opts for a ‘re-vision’ of science that overcomes the traditional dualisms of epistemic subject and object as well as of nature and culture (science). Beyond scientific realism and radical social constructivism, Haraway understands ‘nature’ or ‘world’ neither as a passive resource nor as a human product of imagination. Rather, she argues, the world is to be understood as a ‘witty agent’ that has its own efficacy and historicity in the production of knowledge. Instead of epistemic reification, possession, and appropriation of ‘nature’, knowledge production should be understood as a conversation between material-semiotic actors, human, and non-human, from which none of the actors leaves as they entered. In this study, I want to explore what it means to conceive of nature or world in knowledge processes as a “witty agent” and how exactly one is to imagine this form of non-human agency. To this end, I will first explain Haraway’s re-vision of “nature” beyond scientific realism and radical social constructivism (sect. 2). From this, I will discuss her underlying conception of agency (sect. 3). This involves first, a reconception of the traditional relation between epistemic subject and object as dynamic and situational relation (sect. 3.1). Second, Haraway characterizes the world’s epistemic agency in more positive terms by using the ‘trickster’ figure as it appears in Southwest Native American representations in the form of a Coyote (sect. 3.2). Finally, I will come back to Haraway’s initial question of an objective scientific approach to the world, which for her consists in a power-charged social relation of conversations with the world. I will conclude with a critical reflection of what Haraway’s conception of the world as an agent means for scientific practice and its engagement with objects of knowledge.

## 1 Introduction: Haraway’s theorem of “situated knowledges”

Donna Haraway’s writings on philosophy of science revolve around the fundamental interweaving of the practices and conceptions of knowledge and politics, analyzing the complex interdependencies from ontological, epistemological, linguistic-semiotic, material, and aesthetic aspects. Her influential essay “Situated Knowledges: The Science Question in Feminism and the Privilege of Partial Perspective” (1988) occupies a central position here, in which she explores the question of how we are to understand the “curious and inescapable term ‘objectivity’” (SK^1^, p. 183) from a feminist perspective. Originally written as a commentary on Sandra Harding’s The Science Question in Feminism (Harding, 1986), she argues for a revision of the traditional concept of objectivity as a ‘view from nowhere’ in favor of an embodied objectivity and situated knowledges, that is, a ‘view from somewhere.’^2^

Since then there have been quite a few philosophers of science who (with or without reference to Haraway) support a ‘situatedness of knowledge’ and a ‘view from somewhere’ (*cf.* exempl. Harding, 1991, pp. 11 f.; Code, 1995; Barad, 2007, Ch. 8; Elgin, 2017, pp. 157–159; Stephen, 2021, Ch. 4.2) and even declared ‘situated knowledge’ a “paradigm” of feminist critique of science (*cf.* Singer, 2005, p. 29). Yet, especially in more “mainstream,” that is, not decidedly feminist, philosophy of science, it is often neglected that Haraway’s approach of a situated knowledge does not simply have the rather “flat” meaning of a local and historical localisation of knowledge (Haraway and Goodeve, 2018, p. 71).^3^ Rather, Haraway answers the question of objectivity of the sciences, understood both in a semantic (or metaphysical) sense as the question of truth, accuracy, the referential character of scientific theories, and in an epistemological sense as the question of the non-arbitrariness and non-subjectivity of scientific methods, through a fundamental revision of the subject–object relationship in epistemic contexts. Her theorem of situated knowledge as a view from somewhere therefore does not only mean a methodological reflection on “what your identifying marks are and literally where you are” (Haraway, 2000, p. 71), in order to avoid the illusion of a divine view from nowhere. On an *epistemological* level, the theorem of situated knowledge in addition includes a critique of the idea of a ‘neutral’ observer position that supposedly generates unbiased representations of objects of knowledge (*cf.* Hoppe, 2021, p. 63). Finally, on an *ontological* level, Haraway’s essay aims at a revision of both the epistemological ‘subject’ and the ‘object’ of knowledge and science, i.e., the world or nature (*cf.* Hoppe, 2021, p. 86 f.). According to this revision, the world or nature is not to be understood as a passive resource for epistemic access by scientists but as a witty agent that has its own efficacy and historicity in the production of knowledge.^4^

In the following, I will focus on Haraway’s ontological revision of the object of knowledge, which is often neglected in discussions and adaptions of “Situated Knowledges,” that is, I will focus on the semantic or metaphysical level of meaning of scientific objectivity and try to clarify what it means to conceive of nature or world in knowledge processes as a “witty agent” and how exactly one is to imagine this form of non-human agency. Yet, this limitation is a focus on specific aspects of Haraway’s essay “Situated Knowledges,” rather than a strict disciplinary constraint. This is because the subject of scientific objectivity, both in general and within Haraway’s work, does not permit a clear-cut separation between metaphysical-ontological, epistemological, and political aspects. Instead, the concept of *objectivity*, regardless of one’s interpretation, revolves around the intricate overlaps of and tensions within these domains. This is what Haraway’s metatheoretical claim in “Situated Knowledges” means when she says that she “want[s] to argue for a *doctrine* and practice of objectivity” (SK, p. 191): That is, her conception of “objectivity” is to be understood both as a systematic doctrine or guideline with normative claims and as a practice, that is, something we must do and learn.

## 2 Beyond scientific realism and social constructivism: Haraway’s artifactual nature

By committing herself to the goal of finding an answer to the question of scientific objectivity from a feminist perspective, Haraway, on the one hand, wants to avoid the illusion of a divine view from nowhere. But on the other hand, she insists that from a feminist perspective, it is not enough to point out the radical historical contingency and construction of all knowledge—rather, “[f]eminists have to insist on a better account of the world” (SK, p. 187). With regard to the ontological question of scientific objectivity, whether and if so, how science can offer faithful accounts of the world, she claims “to have *simultaneously* an account of radical historical contingency for all knowledge claims and knowing subjects, a critical practice for recognizing our own ‘semiotic technologies’ for making meanings, *and* a no-nonsense commitment to faithful account of a ‘real’ world” (SK, p. 187). In other words, Haraway wants to develop a philosophy of science that overcomes the juxtaposition and thus also the respective shortcomings of realism and (radical) social constructivism in order to arrive at a situated but nevertheless faithful conception of objectivity. The point of contention between realists and (radical) constructivists (or other anti-realists) in philosophy of science can be formulated as follows: Are scientific results *discoveries* of a language- and mind-independent world (or nature) or are they *inventions* by means of which ‘the world’ (‘the nature’) is—at least partially—constructed?

Far from dismissing the question as idle armchair speculation, Haraway takes it as a symptom of the politically powerful dualisms that disrupt Western thought, that is, thinking in binary, hierarchical conceptual oppositions in which one part is conceived as original and superior, the other as derivative of the former and inferior. Examples of these are as follows: True/false, active/passive, reason/feeling, subject/object, human/animal (nature), culture (science)/nature, and man/woman.^5^ While according to the realist view, nature is a passive resource whose properties and laws are discovered and epistemically appropriated by scientists, according to the radical constructivist view, humans produce nature culturally and scientifically, so that beyond human construction it seems to be nothing but a “blank page for social inscriptions” (SK, p. 197). Thus, according to Haraway, both approaches suffer from a “poductionist” way of thinking, especially in relation to nature or the world as an object of knowledge: “Productionism and its corollary, humanism, come down to the story line that ‘man makes everything, including himself, out of the world that can only be resource and potency to his project and active agency’” (PM, p. 67). Such a “productionist logic seems inescapable in traditions of Western binarisms” (SK, p. 198); indeed, it can also be understood as their political significance and problematic.

The political problematic of Western dualisms is that they are not simply disinterested distinctions, so to speak, but relations “of separation and domination inscribed and naturalized in culture” which simultaneously rely on and deny the agency of subordinated Others (Plumwood, 1993, p. 47; *cf.* Plumwood, 2001). More specifically, the dualism between nature and culture (science) that characterizes Western thought inevitably leads to a productionist logic and “typical hegemonic constructions of agency” (Plumwood, 2001, p. 14), in which nature and the social others declared to be nature form the passive resource or foil for man’s (epistemic) agency—be it a discovering or an inventing one. As is well known, this way of thinking has found explicit theoretical expression in “The Great Chain of Being” hierarchy (also known as *scala naturae*). Even without being openly expressed, this hierarchy of ‘nature’ is still operative in numerous forms and various areas, often unconsciously (*cf.* Plumwood, 2001, p. 9). What looked like an innocent or at least purely academic question about the relationship between science and its object of reference turned out to be only the metaphysical outgrowth of a way of thinking which rests on hegemonic constructions of agency, and as a consequence of which nature is appropriated, subjugated, and exploited. And this is precisely the reason why, for Haraway, answering the question of scientific objectivity requires a reconceptualization of the relationship between the subject and object of knowledge, beyond epistemic and political appropriation, subjugation, and exploitation.

As Haraway points out, various efforts have been made in the past, and immense resources expended in order to be able to (re)grasp the ‘essential reality’ of nature—but with, as she writes, “disappointing results” (PM, p. 296):

Efforts to travel into “nature” become tourist excursions that remind the voyager of the price of such displacements—one pays to see fun-house reflections of oneself. Efforts to preserve “nature” in parks remain fatally troubled by the ineradicable mark of the founding expulsion of those who used to live there, not as innocents in a garden, but as people for whom the categories of nature and culture were not the salient ones. Expensive projects to collect “nature’s” diversity and bank it seem to produce debased coin, impoverished seed, and dusty relics. […] Finally, the projects for representing and enforcing human “nature” are famous for their imperializing essences, most recently reincarnated in the Human Genome Project^6^ (PM, p. 64).

Since “nature” is thus at stake in every respect, its mention seems impossible if it does not appear “suitably surrounded by sneer quotes” (Plumwood, 2001, p. 3), the syntactic marking of a pathos of distance, conveying not only superiority and fear of contact but also dominating the surrounded meta-linguistically and rendering it thus semantically incapable of action: In this situation, it is no longer possible to speak *with* nature, but at most *about it*—or to remain silent about it altogether. But instead of silently renouncing “nature,” letting it burn in its expelled situation, as it were, Haraway argues in favor of a revision of nature. Because for Haraway,^7^ nature

is one of those impossible things characterized by Gayatri Spivak as that which we cannot not desire. Excruciatingly conscious of nature’s discursive constitution as “other” in the histories of colonialism, racism, sexism, and class domination of many kinds, we nonetheless find in this problematic, ethno-specific, long-lived, and mobile concept something we cannot do without, but can never “have” (PM, p. 64).

If we want to give up this repressed, othered ‘nature’, but not *a*, or any nature, we have to find a different relationship to nature according to Haraway, one “besides reification, possession, appropriation and nostalgia” (OC, p. 126; *cf.* Trächtler, 2023). We can neither escape phallo(go)centrism by forgetting nor undermine its roots in the hope of encountering an untouched nature and starting anew, as it were from a *tabula rasa* (*cf.* Trächtler, 2023). “Where we need to move,” says Haraway, “is not ‘back’ to nature, but elsewhere, through and within an artifactual social nature” (PM, p. 90). Such a nature is, as Haraway says,

not a physical place to which one can go, nor a treasure to fence in or bank, nor an essence to be saved or violated. Nature is not hidden and so does not to be unveiled. Nature is not a text to be read in the codes of mathematics and biomedicine. It is not the “other” who offers origin, replenishment, and service. Neither mother, nurse, nor slave, nature is not matric, resource, or tool for the reproduction of man (PM, p. 65).

Rather, Haraway’s new conception is an *artifactual* nature, insofar as “nature for us is *made*, both as fiction and fact” (PM, p. 65)—What does that mean? Is Haraway here not opting for a strong social constructivism after all? And does not this convergence of fact and fiction seem contradictory even from a constructivist, anti-realist point of view? After all, a ‘fact’ is generally understood to be something that *is* (in some sense), whereas ‘fiction’ is something that *is not* (*cf.* Trächtler, 2023). So, what exactly does Haraway mean by saying that nature is made as fact and fiction?

First, Haraway reminds us that ‘fact’ and ‘fiction’ already refer etymologically to human action, performance, even human feats (*cf.* PV, pp. 3 f.). Facts are thus not only to be understood as ‘made of’ something found in nature or in the world but always also as ‘made-up’, something invented, constructed, speculative. Likewise, fictions never arise solely from the human mind or imagination, they are never entirely ‘made-up’ but always also ‘made of’ something found in nature or the world that resists the arbitrariness of human projections (*cf.* Trächtler, 2023). Haraway herself gives the example of organisms that are not born, not found or discovered, but are *made*, insofar as “they are not pre-existing plants, animals, protists, etc., with boundaries already established and awaiting the right kind of instrument to note them correctly”—rather, organisms “emerge from a discursive process,” namely biology, which is “a discourse and not the living world itself” (PM, p. 67; *cf.* Trächtler, 2023). And this applies analogously to other rhetorical references to ‘nature’ and ‘naturalness’, such as so-called ‘natural kinds’, which conceal the fact that they are the result of interest-driven processes of creation and classification, which becomes particularly clear in examples such as ‘toxic kinds’ or ‘protists’ (*cf.* Elgin, 2019, pp. 524 f.). Conversely, our scientific and non-scientific fictions are not *purely* invented, not only the product of exclusively human creative processes: The objects of science, such as organisms, cells, sex as objects of biology, cannot be entirely reduced “to the ephemera of discursive production and social construction,” because this would mean losing “the body itself as anything but a blank page for social inscriptions, including those of biological discourse” (SK, p. 197). In this sense, knowledge production is a *material-semiotic* practice in which the discursive and the bodily, material permeate or shape each other (*cf.* PM, p. 67 f.).

Fact and fiction are thus products of a more or less creative process, they are both made but they are not the same (*cf.* PV, p. 3 f.; Hoppe, 2021, p. 98 f.). For a revision of nature beyond reification, possession, appropriation, and nostalgia, that is, beyond the productionist logic that produced the polarization of realism and radical constructivism (*cf.* Hoppe, 2021, p. 98), we must acknowledge both the (discursive) construction in the seemingly ‘naturally given’, that is, the fictional in the factual, and the contribution of nature in the seemingly purely human achievements, that is, the factual, non-discursive in the fictional.^8^ Particularly in contrast to the radically (social) constructivist creation of nature, Haraway’s reconception of nature as *made* means that it should not only be seen as the result of *human* agency:

If the world exists for us as “nature”, this designates a kind of relationship, an achievement among many actors, not all of them human, not all of them organic, not all of them technological. In its scientific embodiments as well as in other forms, nature is made, but not entirely by humans; it is a co-construction among humans and non-humans (PM, p. 66).

For Haraway, *nature* in ‘its scientific embodiments as well as in other forms’ is neither a blank canvas that presents itself without resistance to the projections of human imagination nor a resource, patiently waiting to be deciphered by us: “The world neither speaks itself nor disappears in favor of a master decoder. The codes of the world are not still, waiting only to be read. The world is not raw material for humanization […]” (SK, p. 198). Rather, artifactual nature—the world—as an object of knowledge is a co-construction between humans and non-humans, i.e., it is itself involved in its construction and should therefore not be thought of passively, but “as an actor and agent”, or more precisely: “as *witty* agent” (SK, pp. 198 f.; my emph.).

## 3 The world as witty agent

But what exactly does it mean to conceptualize the world as a ‘witty agent’ in knowledge processes and how are we to conceive of such an agency?

In the social sciences and humanities, it does not seem controversial to conceive of the object of knowledge as agent and as having an influence on the knowledge projects, so that in these disciplines, according to Haraway, “coming to terms with the agency of the ‘objects’ studied is the only way to avoid gross error and false knowledge of many kinds” (SK, p. 198). But how are we to conceive the agency of the world in all the other sciences whose objects are non-human animals or inert matter, of even abstract entities?

Haraway insists that her conception of the world as agent must also apply to “the other knowledge projects called sciences” (SK, p. 198), insofar as they are committed to a “usable, but not innocent, doctrine of objectivity” (SK, p. 190): “A corollary of the insistence that ethics and politics covertly or overtly provide the bases for objectivity in the sciences as a heterogenous whole, and not just in the social sciences, is granting the status of agent/actor to the ‘objects’ of the world” (SK, p. 198). With this view that non-human animals and even non-living entities are granted the status of agents/actors in knowledge processes, Haraway opts for a very broad concept of agency tying in with the ‘Actor-Network-Theory’ founded by Bruno Latour and others. Haraway adapts this theory for her feminist philosophy of science because the Actor-Network-Theory rejects what she sees as the untenable “separation between politics and science, not in order to reduce scientific knowledge to political interests, but in the sense that politics and science, like society and nature, are to be understood as *co-production*” of different actors (Singer, 2005, p. 131; my transl.). According to Actor-Network-Theory, “*any thing* that does modify a state of affairs by making a difference is an actor”—that is, a hammer can be an actor since, as is easy to see, it makes a difference whether one drives a nail with or without a hammer (Latour, 2005, p. 71). ‘Actor’ or ‘agent’ in this broader sense initially means “*participants* in the course of action” (Latour, 2005, p. 71). This participation does not necessarily have to consist of ‘determining’, ‘causing’, or ‘forcing’ the respective action; rather, this agential participation can also mean to “authorize, allow, afford, encourage, permit, suggest, influence, block, render possible, forbid, and so on” (Latour, 2005, pp. 71 f.).^9^ In this sense, an action is always situated in a social *network* in which different actors—humans, non-human animals, things—interact with each other. In a fundamental sense, Haraway shares this view of ‘agency’ when she states: “the world encountered in knowledge projects is an active entity” (SK, p. 198).^10^

This distinguishes her approach from ‘classical’ accounts of agency in analytical philosophy. Starting from the question of the nature of agency or the circumstances under which we speak of ‘agency’ in contrast to mere happening, these ‘classical’ accounts outline agency often as a (mostly, human) subject’s intentional actions which in turn are in some way linked to mental or cognitive abilities or consciousness in general (*cf.* Anscombe, 1957; Davidson, 1963, 1971). Especially when it comes to agency in epistemic contexts, the prevailing discussions on ‘epistemic agency’ center around the question of whether and, if so, to which extent mental acts and dispositions such as ‘believing’, ‘judging’, and ‘inferring’ are actions, so that other and non-human forms of epistemic agency are left out of consideration (*cf.* Ryle, 1949; Sosa, 2015; Ch. 9; Hunter, 2022). However, Haraway’s approach also differs from more recent accounts in philosophy of agency allowing degrees and ‘minimal’ forms of agency, which can also be attributed to non-human animals, organisms, and plants if they exhibit behavior that reveals individuality, a goal-directedness following norms (e.g., self-maintenance) and causal efficacy (*cf.* van Hateren, 2022).^11^ In contrast to these theories of agency in the narrower sense, Haraway is not primarily concerned with the question of the nature of agency or the circumstances under which we speak of ‘agency’ or actions, for instance in contrast to mere happenings. Her theoretical starting point is the question of the meaning of objectivity in a feminist theory of science, which cannot rely on an understanding of ‘knowledge’ as the product of an epistemic subject’s omnipotent agency over a passive object.

Rather, in her inclusion of diverse forms of actors and agency, Haraway is concerned with understanding the social and political structure of epistemic actions in knowledge production beyond hegemonic constructions of agency: That is, for her, all participants—humans, non-human animals, machines, instruments, and matter—in knowledge production have (some) epistemic agency. This conception of agency in epistemic contexts entails that the ontological boundaries between epistemic subject and object are not *a priori* determined and definite: “Situated knowledges require that the object of knowledge be pictured as an actor and agent, not a screen or a ground or a resource, never finally as slave to the master that closes off the dialectic in his unique agency and authorship of ‘objective’ knowledge” (SK, p. 198). In other words, with her reconception of the world as an agent in knowledge production, Haraway wants to transcend the long problematic boundaries between epistemic subject and object, between humans, non-human animals, and technology, and between body and mind. Accordingly, Haraway understands agency as defined by the relationships, connections, or associations of the various actors participating in the course of an action, that is, the actors, agents, are not ‘stable’ entities with well-defined boundaries, but rather constitute themselves mutually and situationally and are thus dynamic and relational. While Haraway’s view thus seems far removed from classical theories of agency, there are significant overlaps with enactivist accounts of agency: Enactivist accounts of agency focus on the relation between an organism and its environment (instead of an individual’s mental states) emphasizing relational and situational interactions between various kinds of actors. Much in line with Haraway, Di Paolo, for example, stresses that knowing is a co-productive activity by which agents “bring forth a world,” in both epistemic and ontological terms, such that knower and known are inseparable (DiPaolo, 2023, esp. pp. 167); and DeJesus combines enactivist approaches with the work of Haraway, Barad, and Latour to defend an onto-epistemological position where “worlds, novel ontological entities and phenomena, are continuously being concretely made—*enacted*—through historically situated, temporally co-evolving webs of entangled relations constituted by a multitude of agents, assorted materials and technologies” (DeJesus, 2018, 880).^12^ What these enactivist approaches have thus in common with Haraway’s account is that ‘(epistemic) agency’ here refers to the way in which humans, non-human animals, instruments, and machines interact with each other and are thus involved in the production of knowledge.

However, if the world’s ‘agency’ is understood in such a broad sense, it seems hardly surprising, but rather trivial, that the ‘non-human’, even dead and abstract parts of the world with which other scientific disciplines are concerned are also granted the status of agents. Moreover, since this view of the world as an agent involved in knowledge processes is a basic ontological assumption, which, as Weber noted, is just as impossible to prove or disprove as realist or (social) constructivist conceptions of the scientific object (*cf.* Weber, 2003, pp. 253 f.), it seems as if one could just shrug shoulders and dismiss such ontological accounts as idling wheels in the philosophical machinery. One wants to say: *Of course*, in addition to the ‘men of science’, their microscopes, laboratory pigs, metal samples and texts also *participate* in the production of knowledge in a certain sense. But is it not a gross, or at least counterintuitive, exaggeration and capricious theoretical complication to elevate all these entities to *agents* in knowledge production?

In other words, what does Haraway want to achieve with her new conception of the world as an agent in knowledge contexts, what advantages does her approach have?

In the following, I will clarify this question with regard to two aspects: First, Haraway’s thesis of active objects of knowledge as agents is to be understood against the background of her reconception of traditional subject–object dualism in epistemic contexts, which for her is not just an ontological presupposition of purely academic interest, but politically and epistemologically effective. Second, I will use Haraway’s specification of the world’s *witty* agency in the form of the ‘trickster’ figure of the Coyote to explain that the agency of knowledge objects exceeds that seemingly trivial notion of agency as mere participation in knowledge processes: According to Haraway, the objects of knowledge really are ‘stubborn’.

### 3.1 Re-vision of the subjects and objects of knowledge

In contrast to the criticism suggesting that Haraway’s characterization of the object of knowledge as an agent is an ontological assumption deserving no further consideration due to its unprovability and non-refutability, I have pointed out earlier (in section 2) how, within the productionist logic underlying realism and social constructivism, the ontological assumption of passive knowledge objects is intertwined with their epistemological appropriation, subjugation, and exploitation and thus politically effective. ‘Nature’ and the social others declared to be nature can be epistemically and politically oppressed by denying them any agency as radically ‘othered’ objects and by degrading them to the passive background of ‘man’s’ agency who thus appear to be omnipotent. In contrast, Haraway’s view has the advantage, as Weber also acknowledges, of avoiding such “phantasies of omnipotence in the same way as determinisms—both in their naturalistic and culturalist versions” (Weber, 2003, p. 254; my transl.). For if one conceives of the object of knowledge as agent participating in the production of knowledge, this also entails a significant redefinition of the relationship to the subject of knowledge (*cf.* Hoppe, 2021, p. 90): If we can still speak of the ‘subject’ and ‘object’ of knowledge at all, then *not* in a modern sense of two distinct, definitively determined and finished entities, one of which serves merely as a passive resource and foil for the epistemic endeavors of the other, but *at most* in the sense of a dynamic situational relationship in knowledge processes.

The knowing self is partial in all its guises, never finished, whole, simply there and original; it is always constructed and stitched together imperfectly, and *therefore* able to join with another, to see together without claiming to be another. Here is the promise of objectivity: a scientific knower seeks the subject position not of identity, but of objectivity; that is, partial connection (SK, p. 193).

According to Haraway, subjects are never coherent, finished, and self-identical entities, which encounter distinct, equally finished objects in knowledge contexts. Rather, the subject, understood as the knowing self, is always heterogeneous, divided, and contradictory (*cf.* SK, p. 193), that is, Haraway here opposes, on the one hand, modern epistemology’s framework of a transparent, disembodied subject of knowledge that faces its object distinctly and disinterestedly. This view is still present in post-positivist-empiricist mainstream epistemology when propositional knowledge claims are represented, for example, as “*S* knows that *p*,” where ‘S’ stands for a human, in principle transparent and thus interchangeable individual, and where ‘p’ stands for a knowable proposition about an object, usually handy physical objects such as apples, pieces of paper, and color patches (*cf.* Code, 1995, pp. 27 f.).^13^ On the other hand, Haraway is also critical of (some) standpoint theories, insofar as these, too, are based on a coherent, finite conception of the subject, except that the epistemic privilege here is not attributed to the transparent and disembodied subject position of ‘inappropriate/d others’ (*cf.* Minh Hà, 1986/1987). According to Haraway, the knowing self is neither a disembodied, unbiased, and all-seeing mind, nor is the knowing self simultaneously in all or completely in one of the oppressed positions as structured by gender, race, nation, and class (*cf.* SK, p. 193), that is, a scientist can, for example, be oppressed as a woman, but at the same time be dominant as a white, central European, member of the upper class. According to Haraway, subjects of knowledge are thus always heterogeneous, fractured in themselves, and their boundaries are porous—which is precisely why they can enter into partial connections with other objects or actors involved in the production of knowledge (*cf.* Hoppe, 2021, pp. 78 f.).

This blurring of the subject–object boundary becomes clearer if we look at the role of optical devices in scientific practice, such as cameras, microscopes, models, but also organic eyes:

The “eyes” made available in modern technological sciences shatter any idea of passive vision; these prosthetic devices show us that all eyes, including our own organic ones, are active perceptual systems, building in translations and specific ways of seeing, that is, ways of life. There is no unmediated photograph or passive camera obscura in scientific accounts of bodies and machines; there are only highly specific visual possibilities, each with a wonderfully detailed, active, partial way of organizing worlds (SK, p. 190).

Etymologically and philosophically, ‘seeing’ was always linked to ‘knowing’ in dominant Western languages and philosophical traditions. Haraway re-appropriates this link and emphasizes that first, the ability to see always requires mediation by a body—be it a technological device or an organ (*cf.* SK, p. 190). Second, such seeing is always bound to a specific, particular perspective from which something is partially seen, which at the same time also situates the seeing agent in a specific location. In this sense, our visual technology can be understood as a political and epistemological ‘si(gh)ting device’ (*cf.* SK, pp. 190, 201). Third, seeing is a practice that must be learnt: Organically as well as technologically mediated seeing always involves ‘not seeing (disregarding) something’, ‘seeing something *in* something’, and ‘seeing something *as* something’, that is, seeing involves different, active, and partial possibilities “of organizing worlds” (SK, p. 190). Understood as such a bodily mediated and learnt practice, seeing does not provide undistorted representations of its objects, no reflections, but rather *diffractions*: Diffraction is the deflection of waves at an obstacle, causing them to overlap, combine, and spread out. In this way, “[d]iffraction does not produce ‘the same’ displaced, as reflection and refraction do. Diffraction is a mapping of interference, not replication, reflection or reproduction. A diffraction pattern does not map where difference appear, but rather maps where the *effects* of difference appear” (PM, p. 300). In these diffractions as, for example, created by seeing with optical devices, it becomes clear how embodied seeing and what is seen overlap and how diffraction patterns produce each other, so that subject and object in knowledge production exist as a situational and dynamic reciprocal relationship (*cf.* Prins, 1997, Ch. 4).^14^

In her reconceptualization of the knowledge objects as an active entity, Haraway *on the one hand* points out that the rigid dichotomy between a definitively fixed subject position occupied by an active human scientist and an object position occupied by a passive ‘natural’ object is a (philosophical, theoretical) fiction (*cf.* OC, p. 126). In scientific practice—at the latest in technoscientific times of the “convergence of biotechnologies, information technologies, and nanotechnologies” (Barad, 2007, p. 27)—the boundaries associated with subject-object dualism between organisms and technology, human and non-human, mind and AI, reproduction and replication have long been problematic, if not obsolete (*cf.* Singer, 2005, p. 134 f.). However, the subject–object dichotomy is only *one* ‘onto-epistemological’ conceptualization in Pandora’s Box of Western dualisms. In addition to a blurring of the subject–object boundary, Haraway’s reconception of knowledge objects as agents, *on the other hand*, is also about a re-vision of the dualism between (bodily-passive) nature and (mental-active) culture/science. As I will show in the following, Haraway’s conception of the world’s agency thus goes beyond a mere *participation* of the world in knowledge production, in the sense of Latour’s earlier mentioned hammer-actors, insofar as Haraway characterizes the world as a ‘*witty* agent’.

### 3.2 The coyote as witty trickster

Characterizing her reconception of the world as *witty* agent, Haraway uses the ‘trickster’ figure as it appears in Southwest Native American representations in the form of a Coyote: here, as in other stories and mythologies, ‘trickster’ means a hybrid being between god, human, animal, and spirit, that is, a metaphysical border-crosser, which eludes ontological definition (*cf.* Hynes, 1997, p. 33). Tricksters can benefit humans, but at the same time, they are also prowlers and crooks; beyond good and evil, they are masters of transformation who ‘reshuffle the cards’ and change situations. Within creation myths, both European tricksters and the Coyote trickster in Southwest American Navajo^15^ mythology play the role of cultural heroes who reveal (sometimes divine) knowledge of cultural techniques to humans (*cf.* Cooper, 1987, p. 184). Haraway appropriates the Coyote as a trickster figure, while she is critically aware that her use of this figure “is marked by middle-class, white feminist appropriation of Native American symbols, about which one must be very suspicious,” as it often operates “in a rather colonial way to Native American practices” (Haraway, 2004, p. 327). Under this sign of caution, however, she also notes that “figures do travel, and they travel outside of their places of emergence in various ways,” so that the influence of Native American symbols is not limited to Native culture—“and who is to say that Native American symbols are to be less global than those produced by Anglo-American” (Haraway, 2004, p. 327 f.)? It is precisely the fact that such figures can transcend their origins in unexpected, surprising ways which, according to Haraway, makes them “politically interesting, although certainly not innocent”:

Thus, the coyote is a specific figuration. It is not nature in a Euro-American sense and not about resources to the makings of culture. Moreover, coyote is not a very nice figure. It is a trickster figure, and, particularly in Navaho figurations, the coyote is often associated with quite distressing kinds of trickster work. Coyote is about the world as a place that is active in terms that are not particularly under human control, but it is not about the human, on the one side, and the natural on the other. There is a communication between what we would call “nature” and “culture”, but in a world where “coyote” is a relevant category, “nature” and “culture” are not the relevant categories. Coyote disturbs nature/culture ontologies (Haraway, 2004, p. 328).

By using the coyoteas a “visualization” (SK, p. 199) for the world or nature in knowledge processes, Haraway employs a figure that encourages us—that is, all those who are held captive by Western dualisms—to rethink our relationship to nature in knowledge processes *beyond* the Western nature-culture dualism. Understanding the world as a coyote in knowledge contexts does not only imply a *mediation* between ‘nature’ and ‘culture’, but that these are simply not relevant as categories, precisely *because* culture/knowledge is never solely the product of human agency and, conversely, ‘nature’ or ‘world’ as object of knowledge is never a naked, untouched thing in itself but is always preformed by theories, methods, apparatuses of perception, interests, and social factors. If the object of knowledge actively appears in the form of a Coyote-like *culture hero*, for example, these situations cannot be meaningfully conceptualized in the conventional categories of ‘nature vs. culture’.

Visualising the object of knowledge as Coyote which actively participates in knowledge processes also means that it “may operate in ways that humans cannot predict or control” (McAlister, 2010, p. 133). However, this is not due to a ‘lack’ or inability on the human side—rather the *wittiness* of the world lies in the fact that it can also *evade* epistemic access, even stubbornly resist it. Haraway thus emphasizes that the object of knowledge has a stubbornness, which admits “some unsettling possibilities, including a sense of the world’s independent sense of humor” (SK, p. 199). Feminist objectivity, which takes this stubbornness seriously, therefore also means to make “room for surprises and ironies at the heart of all knowledge production; we are not in charge of the world” (SK, p. 199).

Haraway herself has emphasized these trickster qualities of an active object of knowledge in her academic home, primatology (*cf.* in particular *Primate Visions: Gender, Race, and Nature in the World of Modern Science*, 1990).^16^ Nevertheless, the world’s witty agency as stubbornness, resistance, and evasion is not limited to living and organic objects of knowledge; rather, inorganic matter, non-material phenomena, and artifacts such as texts, images, and models are also ‘witty’, stubborn, and can resist epistemic access (*cf.* Hoppe, 2021, 181 f.). For example, Haraway worked out nature’s trickster qualities by examining the various representational practices of ‘nature’ in scientific images and models (*cf. Modest_Witnesses@Second_Millenium*, 2018), advertisements (*cf.* “Promises of Monsters: A Regenerative Politics for Inappropriate/d Others,” 1992), and museums (*cf.* “Teddy Bear Patriarchy: Taxidermy in the Garden of Eden, New York City, 1908–1936,” 1984–1985 [in PV]). However, in modern physics, the stubbornness and the resistance of its rather ‘dead’, modeled and non-material objects of knowledge, together with the implications for the knowing subjects and the technologies and methodologies used, become particularly evident.

Barad (2007) has impressively demonstrated this ‘entanglement’ of nature and culture, subject and object, the discursive and the material in relation to quantum physics. One of the examples that she cites in the course of this, which at the same time sums up Haraway’s characterization of nature as a witty trickster *and* the multi-layered, ironic ‘situatedness’ of knowledge, is the Stern–Gerlach experiment. This experiment was carried out by Otto Stern and Walther Gerlach in Frankfurt in 1922 to demonstrate directional quantisation: At that time, physics was characterized by scientific uncertainty, which the then new quantum ideas meant for classical physics (*cf.* Barad, 2007, p. 162). Stern and Gerlach hoped that the successful realization of the experiment would lead to a clear decision between quantum theoretical and classical views (*cf.* Barad, 2007, p. 162). A much-discussed question was whether the phenomenon of so-called ‘directional quantisation’, that is, the fact that the orientation of the electron orbit is only limited to discrete, specific spatial values, was a real phenomenon or merely the symbolization of another phenomenon that was not yet understood (*cf.* Barad, 2007, p. 162). With their experiment, Stern and Gerlach intended to prove that, contrary to the majority’s opinion, directional quantisation was a real phenomenon, and they wanted to demonstrate this by dividing a beam of electrically neutral silver atoms into two parts using a specific arrangement of magnets, which would leave two separate tracks on a glass plate (*cf.* Barad, 2007, p. 163). Despite the successful setup and correct execution of the experiment, the traces of directional quantisation did not reveal themselves when *Gerlach* inspected the glass plate—but as *Stern* approached it, strangely enough, they suddenly appeared. As it turned out, this was due to the seemingly random circumstance that Stern was smoking cigars at the time, namely *cheap* cigars due to his low salary, which, unlike good cigars, contained a lot of sulfur, so that his “breath on the plate turned the silver into silver sulfide, which is black, so easily visible” (Friedrich and Herschbach, 1998, p. 178 f.; cited after Barad, 2007, p. 164). Barad adds a schematic representation of the experiment to her discussion of it (*cf.* Barad, 2007, Figure 15), which also contains the cigar that is usually not shown, explaining it as follows:

A next-order iteration of the schematic of the Stern-Gerlach experiment, revised to more accurately account for the nature of the apparatus. This schematic includes the crucial agential contribution of the cigar. The reproducibility of the experiment depends on the cigar’s presence. Not any old cigar will do: the high sulfur content of a cheap cigar is crucial. Class, nationalism, gender, and the politics of nationalism, among other variables, are all part of this apparatus (which is not to say that all relevant factors figure in the same way or with the same weight) (Barad, 2007, p. 165).

Barad’s modified figure really shows a diffraction pattern rather than a representation (in the narrower sense), insofar as it maps the interference (of the cheap cigar) that effected the decisive difference between the experiment’s failure and success. The sheer randomness and arbitrariness of the experiment’s success compared to its careful and painstaking set-up, which required several attempts and days, was later attributed to the “uncanny conspiracy of nature” (*cf.* Friedrich and Herschback, 2003; cited in Barad, 2007, Ch. 4, note 53), to which Barad notes:

These are curious conclusions to draw about Stern and Gerlach’s complex intra-actions with nature. It seems as if the authors could just as easily (if not more justifiably) have paid homage to nature for being so remarkable cooperative in presenting a productive coincidence rather than a null result (Barad, 2007, Ch. 4, note 53).

In contrast to an agency in the sense of an “uncanny conspiracy of nature,” Haraway’s figure of nature as witty trickster, coyote, points to “our situation when we give up mastery but keep searching for fidelity, knowing all the while we will be hoodwinked” (SK, p. 199). The Stern–Gerlach experiment shows this situation all too clearly: the boundaries between subject, object, and experimental apparatus, as well as the boundaries between ‘passive nature’ and ‘active culture’ are blurred, the knowledge produced is a co-production of people, cigars, and atoms, and its success depended largely on contextual factors classically regarded as ‘contingent’, ranging from inflation in Germany to the additives in cigars and the lack of smoking restrictions at the time to personal (and gendered!) vices (*cf.* Barad, 2007, p. 164). This is less to be blamed on a ‘conspiracy of nature’ than on the “surprises and ironies at the heart of knowledge production,” which, according to Haraway remind us that ultimately, “we are not in charge of the world” (SK, p. 199). And Haraway attempts to do justice to this with her re-vision of the culture-nature relationship and the associated subject–object relationship in knowledge processes, as one in which the world appears as a “trickster with whom we must learn to converse” (SK, p. 201).

Haraway’s philosophy of science is thus not to be understood as an appeal to eliminate or radically overturn all previous practices, discourses, methods and bodies that characterize our knowledge projects. Her, as she says, “simple, perhaps simple-minded manoeuvre” (SK, p. 199) consists rather in this re-vision of traditional dualisms in the production of knowledge which fixes the object of knowledge in the passive position of a resource or screen opposed to the phantasy of human omnipotence, thus evoking the controversial question between realism and radical social constructivism as to whether this omnipotent human subject now discovers or invents the passive ‘natural objects’. According to Haraway, no ‘objective’, that is, faithful *and* responsible knowledge can be achieved on such an onto-epistemological basis; instead, the agency and autonomy, the historicity, and effectiveness of nature must be taken seriously and acknowledged. Objective scientific approaches to the world thus do not depend on a logic of ‘discovery’ or ‘invention’ but on a power-charged social relation of ‘conversation’ (SK, p. 198).

## 4 Sciences as conversations with world or: who speaks for whom?

Haraway’s ontological reconception of the knowledge object as a witty agent implies that knowledge production can neither be grasped realistically as the discovery of the world as a passive resource nor constructivistically as the invention of the world as an empty foil but as a conversation *with* the world, with nature. In conclusion, I will discuss this characterization of science as conversation and, above all, critically point to some limitations of Haraway’s approach.

First, an important aspect of Haraway’s view of science as a conversation is the fundamental lack of closure, in the sense of openness and disputability, of such conversations, because in her eyes, this is precisely what constitutes the objectivity of science as “positioned rationality”:

So science becomes the paradigmatic model, not of closure, but of that which is contestable and contested. […] A splitting of senses, a confusion of voice and sight, rather than clear and distinct ideas, becomes the metaphor for the ground of the rational. We seek not the knowledges ruled by phallogocentrism (nostalgia for the presence of the one true Word) and disembodied vision. We seek those ruled by partial sight and limited voice – not partiality for its own sake but, rather, for the sake of the connections and unexpected openings situated knowledges make possible. Situated knowledges are about communities, not about isolated individuals. The only way to find a larger vision is to be somewhere in particular. The science question in feminism is about objectivity as positioned rationality […,] views from somewhere (SK, p. 196).

With regard to the objectivity of science, such situated knowledge has the advantage of avoiding both absolute or totalitarian knowledge claims and any relativist equalisation of all positions: “Relativism and totalization are both ‘god-tricks’ promising vision from everywhere and nowhere equally and fully […]. But it is precisely in the politics and epistemology of partial perspectives that the possibility of sustained, rational objective enquiry rests” (SK, p. 191). As Haraway repeatedly emphasizes, the partial perspectives in situated knowledges differ from relativism in that “not just any partial perspective will do” (SK, p. 192): Rather, critical, trained, flexible perspectives are needed, and we are also “bound to seek perspective from those points of view which can never be known in advance” (SK, p. 192).^17^ In this way, science produces “partial, locatable, critical knowledges sustaining the possibility of webs of connections called solidarity in politics and shared conversations in epistemology” (SK, p. 191).

As is already implied here, Haraway’s conception of science as a conversation also means, above all, understanding it as intrinsically socially constituted. As socially structured, science is always characterized by power relations, and these can be better taken into account in the context of a ‘conversation’ between different agents than in the context of a one-sided, seemingly omnipotent human creation/production. This is why Haraway believes, as stated earlier, that objective—faithful and responsible—approaches to a ‘real’ world are not a matter of discovery or invention but depend on a “power-charged social relation of ‘conversation’” (SK, p. 198). Objective, and thus rational, knowledge cannot consist in denying these power relations in a relativistic or absolutist manner but in conducting a “power-sensitive conversation” with the agents involved (SK, p. 196). In other words, science as a power-sensitive conversation means abandoning one-sided, monological accesses to a passive world and instead understanding it as a dialogue or polylogue, as reciprocal interrogations between human *and* non-human material-semiotic actors, from which none of the participants leaves as they entered and which thus bring about differences—epistemic, ontological, and political differences—in the world (*cf.* Hoppe, 2021, pp. 91–93).

But in what sense can we say that the world ‘speaks’ in knowledge projects? Haraway is obviously not saying that human scientists must begin to *chat* with the objects of knowledge—be they people, cells, metal samples, or abstract relations. As a *conversation* between human and non-human agents, Haraway understands science, the production of knowledge, “in terms of articulation rather than representation” (PM, p. 89). The non-human “[n]ature [may] be speechless, without language, in the human sense; but nature is highly articulate” (PM, p. 106). Language, like bodies, is not a precondition but an effect of articulation, that is, concepts and bodies as well as their boundaries are still being negotiated in knowledge processes (*cf.* PM, p. 106) and can therefore not be regarded as given or final, but are always provisional, unfinished, disputable (*cf.* PM, p. 89). This creates space to also acknowledge those agents and their bodies of knowledge in science who, according to traditional understandings, have no agency, such as ‘nature’ and the social others declared to be nature: And it is precisely in “acknowledging the agency of the world in knowledge” (SK, p. 199) which one might call the ontological core of “Situated Knowledges.”

However, *acknowledgment* is a complex matter. And here, where Haraway speaks of “acknowledging the agency of the world in knowledge,” it raises the question of whether this does not lead back to a productionist logic in which an ultimately passive nature requires acknowledgment by the omnipotent agency of (hu)man (*cf.* Singer, 2005, p. 134). However, I do not understand Haraway here to mean that acknowledgment is ‘necessary’ in order for nature or world to have agency in the first place (*cf.* Singer, 2005, p. 134; *cf.* also Hoppe, 2021, pp. 94 f.): Although Haraway’s phrasing cited above is ambiguous in this respect, she clearly states elsewhere that “the world encountered in knowledge projects *is* an active entity” (SK, p. 198; my emph.). In addition, a humanly *conferred* agency would also contradict her characterization of the world’s agency in the form of the witty, stubborn coyote. Haraway emphasized the relationship between human and non-human agents more clearly in her essay “Promises of Monsters”:

Actors are entities which do things, have effects, build worlds in concatenation with other *unlike* actors. Some actors, for example specific human ones, can try to reduce other actors to resources – to mere ground and matrix for their action; but such a move is contestable, not the necessary relation of “human nature” to the rest of the world. Other actors, human and unhuman, regularly resist reductionisms. The powers of domination do fail sometimes in their projects to pin other actors down; people can work to enhance the relevant failure rates. Social nature is the nexus I have called artifactual nature (PM, p. 86).

This shows that the acknowledgment of the agency of non-humans by humans is not *ontologically* necessary; rather, agents are all those “entities which do things, have effects.” It is therefore not a matter of acknowledging the objects of knowledge *as* agents but rather the effects of this agency ‘*in knowledge*’. Nature, the world, the objects of knowledge have agency, independent of humans, but this has been and is often oppressed, marginalized, or pushed into the background in epistemic and other contexts. Sometimes, however, this oppression fails, and we should work on enhancing such failure rates. This is exactly why we need a re-vision of nature as *artifactual* nature.

With her redescription of nature *as* an agent as opposed to the prescriptive appeal to *elevate* nature to an agent, Haraway decidedly ties in with ecofeminist views (*cf.* SK, p. 199) and makes them fruitful for a theory of science. In the course of her ‘progressive naturalism’, Plumwood has formulated various critical strategies to acknowledge these active contributions of nature and deconstruct oppressive dualisms (*cf.* Plumwood, 2001). According to Plumwood, both *naturalisation* and *denaturalisation strategies* are needed to identify what Haraway describes as the interweaving of fact and fiction in nature as a co-construction of human and non-human agents^18^: *Naturalisation strategies* are needed in cases of “deceptive humanness,” that is, where the active contributions of nature have so far not been acknowledged but repressed into the background, such as “[c]ounting something (e.g., a place) as purely human when it involves the labor of nature jointly with human labor,” which “can hide or deny the logical dependency relations in that construction” (Plumwood, 2001, p. 19). However, *denaturalisation strategies* are needed in cases of “deceptive naturalness,” that is, where something is regarded as “purely natural” or has been “naturalized,” when it is actually a human or social (co-)construction, “often in the interests of making it seem unchangeable, of appropriating it,” for example, when gender oppression is justified by “woman’s nature” (Plumwood, 2001, p. 19). A *combination of naturalisation and denaturalisation strategies* is needed when certain human–social relationships and contributions are hidden *by* counting “the human groups involved themselves as nature,” so that “their contributions will not need to be credited or noticed” (Plumwood, 2001, p. 20). Plumwood herself gives the example of Australia, which was seen as ‘terra nullius’, the land of no one, open to appropriation because indigenous people were counted as semi-animal ‘nomads’, and their ecological agency in and attachment to the land were discounted (Plumwood, 2001, p. 20). However, the same also applies to knowledge production in the narrower sense, for example, when indigenous knowledge about the healing properties of plants is appropriated, monopolized through patents, and marketed here as a “gift of nature,” as is the case with the medicine Pelargonium (also known as ‘Umckaloabo’/‘Zucol’)^19^: Pelargonium is a plant indigenous to areas of South Africa, widely used by traditional healers of the Zulu, Basuto, Xhosa, and Mfengi tribes to treat different diseases, such as colds, diarrhea, infections of the respiratory tract, and others (*cf.* Taylor et al., 2005). Its Western use to treat tuberculosis is traced back to the Englishman Charles Henry Stevens in 1897 who was treated by a tribal healer with an extract from the roots of Pelargonium and sold the Kapland-Pelargonium to Europe under the name “Stevens’ Consumption Cure” (*cf.* Taylor et al., 2005). Today, Pelargonium is marketed under the brand name “Umckaloabo” in Europe and “Zucol” in the United States, under license from Schwabe (*cf.* Taylor et al., 2005). In 2008, the Alice Community in the Eastern Cape of South Africa contested European patents held by the German company Schwabe as being illegitimate and illegal monopolization of genetic resource from South Africa and the traditional knowledge of the communities in the Eastern Cape Province (*cf.* EPO, 2008). In 2010, the European Patent Office revoked one patent, whereupon Schwabe also stopped pursuing other patents they held on Pelargonium (*cf.* Groenewald, 2010).

The history of Pelargonium can thus be seen as *one* example where the agents involved in knowledge production actively resisted epistemic, political, and economic exploitation through ‘backgrounding’ (*cf.* Plumwood, 2001, pp. 13 f.) and where their agency in knowledge production was acknowledged—at least *qua* patent law. At the same time, this case also shows that such acknowledgment requires mediation by spokespersons and institutions, such as the Alice Community and the European Patent Office, and thus a certain form of representation which Haraway criticized. Advocating representation in this sense can also have empowering potential by making something *present again* (lat. *re-praesentare*) which has been repressed into the background, made invisible, forgotten, such as Barad’s representation of the cigar in the Gerlach-Stern experiment. Furthermore, the Pelargonium case shows that while acknowledgment *can be* accompanied by epistemic, political, and material consequences (such as the consequences attached to patent law in this case), it is also clear that in many, if not most cases, the possibility of acknowledging, in particular, non-human contributions to knowledge processes remains limited to—at most—a symbolic act, which seems pretty useless for non-human actors: If one thinks of laboratory animals and other living, non-human models in knowledge production, acknowledging their participation with “life and limb” (e.g., in the form of a mention in the acknowledgments when the results are published) would seem just as cynical as its concealment is exploitative. Although Haraway *raised* this question in later essays, she did not adequately discuss it either in terms of its ethical problems or in terms of concrete, politically and epistemically effective possibilities for acknowledging such non-human contributions (*cf.* M_W, esp. Part II; WSM, Ch. 3). Referring to the genetically modified and patented “OncoMouse™^20^” in *Modest_Witness*, she does critically analyze the exploitative structures of a knowledge production that “invents” a living being specifically for cancer research, that is, genetically modifies it, patents it, and sells it as a trademark, and thus not only expropriates the life and work of the animal but also exploits its knowledge (*cf.* M_W, pp. 79–101). As a model substituting for a human body, oncomouse is turned into a sacrifice with a secularized but Christian-connoted salvation promise: “s/he suffers, physically, repeatedly, and profoundly, that I and my sisters may live” (M_W, p. 79). Haraway has dealt with the suffering of laboratory animals and question of their agency in particular in *When Species Meet.* She is not of the opinion that the use, or even the killing, of laboratory animals for research purposes is morally impossible but she believes that

We must take non-innocent responsibility for using living beings in these ways and not to talk, write, and act as if OncoMouse™, or other kinds of laboratory animals, were simply test systems, tools, means to brainier mammals’ ends, and commodities. Like other family members in Western biocultural taxonomic systems, these sister mammals are both us and not-us; that is why we employ them (M_W, p. 82).

The problem is actually to understand that human beings do not get a pass on the necessity of killing significant others, who are themselves responding, not just reacting. In the idiom of labor, animals are working subjects, not just worked objects. Try as we might to distance ourselves, there is no way of living that is not also a way of someone, not just something, else dying differentially (WSM, p. 80).

Instead of using oncomice and other laboratory animals as tools and selling them as products, Haraway pleads for acknowledging them as cooperative co-workers and epistemic agents in these knowledge processes, but it remains a mere plea, an appeal, without it being clear how this should be implemented in scientific practice (*cf.* M_W, pp. 199 f.). The oncomouse may *participate* in knowledge processes, s/he may change and possibly improve our research but even if that would somehow be acknowledged—in what sense can one meaningfully speak of an *actual*, epistemically and politically effective, *agency* here, let alone the agency of a ‘witty trickster’? In reality, OncoMouse™ is so far in the background that s/he can no longer even be called an actor *on* the picture. In the eyes of the company that marketed oncomouse, s/he is in fact nothing more than a mere canvas for their almighty projections of genetic modification, that is, the mere material basis, even behind the ‘picture’s’ background: “We do ‘custom-tailer’ mice. We view them as the canvas upon which we do these genetic transplantations” (Schrage 1993: 3D, cited after M_W, 98). Against this backdrop, how could a non-oppressive, non-exploitative research as ‘conversation’ *with* oncomice and other laboratory animals look like? What does it mean to take “non-innocent responsibility for using living beings in these ways”?

Haraway’s views have been heavily criticized and attacked by animal rights advocates (for a discussion of these, *cf.* Wirth, 2015). Weisberg, for example, says: “In reality, animals in labs are not workers—not even alienated workers—but worked-on objects, slaves by any other name. To call them anything else is to gloss over the brutal reality of the total denial of their ability to act in any meaningful way—namely, as self-determining subjects” (Weisberg, 2009, p. 37). In Haraway’s defense, one must say that this criticism overlooks the fact that Haraway’s objectives and perspectives in “Situated Knowledges” and the related following writings are of a scientific-theoretical nature. That is, despite their emphasis on the intrinsic entanglements of onotological-epistemic and political aspects, ethical and moral considerations are not at the center here (*cf.* Wirth, 2015, 127 f.). At the same time, the criticism is justified, *insofar* as it concerns problematic consequences of Haraway’s reconception of the object of knowledge as an agent. Haraway’s emphasis on acknowledging the agency and participation of non-human agents in knowledge processes does indeed harbor the danger of a productionist logic, but, so to speak, in reverse (*cf.* Plumwood, 1993, p. 33 f.): If the world, nature, is *only* seen as active, co-productive, stubborn, and resistant, this tends to level out its passive, vulnerable, endangered side, which needs protection, (institutional) spokespersons, and advocating representation: “Is,” as Hoppe asks, “‘nature’ not also something inert, destroyed” (Hoppe, 2021, p. 95; my transl.)?

Haraway’s reconceptualization of science as conversations with the world as witty agent is an important instrument on a theoretical level for rethinking scientific objectivity beyond epistemologically, ontologically, and politically problematic dualisms, and it is a powerful tool for breaking through ingrained knowledge (*cf.* Hoppe, 2021, p. 176). However, it remains questionable to what extent her revision—in its overemphasis on the world’s or nature’s activity—goes too far and, above all, neglects the necessity of *some* forms of representational and (institutional) spokespersons—especially for non-human actors—in the bureaucratic apparatus of scientific practice. For, “[k]nowledge is in the end based on acknowledgment” (Wittgenstein, 1998, no. 378), says Wittgenstein. And this can be read both in the sense he intended, that knowledge claims have to be acknowledged by a community with certain epistemic and scientific standards to count as ‘knowledge’ in the first place, as well as the other way round, so to speak, in the sense that “in the end” it is only *knowledge* as the product of active contributions and of actual agency that can be acknowledged at all. In the bureaucratic apparatus of scientific practice, there is neither logically nor practically a place for acknowledging negative contributions, especially from non-human actors, as their “silence” or “refusal” (*cf.* Singer, 2005, p. 244). To reinterpret the death of non-human animals as their “refusal to live when their cooperation is utterly disregarded in an excess of human engineering arrogance” (WSM, pp. 72 f.) is a rhetorical trick, and a cynical, dangerous one at that. In fact, the only place of ‘acknowledgment’ that exists for the inert, the destroyed and the dead, lies outside the sciences in memorials and monuments, that is, symbolic representations of those who in fact cannot or can no longer speak for themselves.

## Data availability statement

The original contributions presented in the study are included in the article/supplementary material, further inquiries can be directed to the corresponding author.

## Author contributions

JT: Writing – original draft.

## Footnotes

^1^“Situated Knowledges.” I use abbreviations for Haraway’s works, which are listed in the references.

^2^She does *not* mean that practising scientists have actually committed themselves to such a concept of objectivity as a ‘view from nowhere’, but rather that non-scientists and especially philosophers are “[t]he only people who end up actually *believing* and, goddess forbid, acting on the ideological doctrines of disembodied scientific objectivity” (SK, p. 184).

^3^In an interview, Haraway herself lamented the flat reading of “Situated Knowledges”: “Sometimes people read ‘Situated Knowledges’ in a way that seems to me a little flat; i.e. to mean merely what your identifying marks are and literally where you are. ‘Situated’ in this sense means only to be in one place.” (Haraway and Goodeve, 2018, p. 71). This is not to deny that there are important exceptions where the theorem of “situated knowledges” has been taken more seriously, such as the work of Weber (2003), Barad (2007), and Hoppe (2021), to mention just a few philosophers of science, as well as those opting for “Situated Knowledges” in other fields, such as Braidotti (2018) work on posthumanism. I thank an anonymous reviewer for pointing this out to me.

^4^Weber has pointed out that Haraway uses the terms ‘nature’ and ‘world’ largely synonymously in order to avoid understanding nature solely as the living, ‘natural’ environment of humans, thus rendering the made, constructed character of nature invisible (Weber, 2003, p. 266, note 52). Hoppe shares this view of the broadly synonymous use but adds that this synonymous use could also have strategic reasons for Haraway, since ‘world’, unlike ‘nature, does not carry its dualistic other—‘culture’—with it (Hoppe, 2021, p. 65, note 23). I share Hoppe’s view, especially regarding “Situated Knowledges,” where Haraway *tends* to speak of ‘world’ when she refers to the object of knowledge beyond dualistic distinctions. Following Haraway’s usage, I also use ‘nature’ and ‘world’ largely synonymously in the following, with a tendency toward ‘nature’ when referring to the object of knowledge in a dualistic conception (e.g., sect. 2) and toward ‘world’ when referring to the object of knowledge beyond a dualistic conception (e.g., sect. 3).

^5^These dualisms of Western thought have been scrutinized by many feminist philosophers and other critical thinkers and identified as the root of phallocentric thinking and oppressive hierarchies. A particularly thorough analysis can be found in Plumwood (1993), who also characterises these dualisms as the ‘logic of colonisation’ (*cf.* Plumwood, 1993, Ch. 2), tracing them back via Descartes to Plato. It should be noted, though, that neither Plumwood nor Haraway criticize the distinctions, e.g., man/woman, as such, but their *dualist* construction.

^6^The Human Genome Project was an international research project from 1990 to 2003 with the aim of ‘decoding’ human DNA (the genome). According to their own statements, the “Human Genome Project’s signature accomplishment” was to “generate the first sequence of the human genome” to provide “fundamental information about the human blueprint” and improve knowledge of human biology and the practice of medicine (The Human Genome Project, 2014). The project had thus begun two years before Haraway’s publication of “The Promises of Monsters” (PM) cited above.

^7^The following discussion of Haraway’s revision of nature (as artifactual nature) can be found similarly in Trächtler (2023).

^8^As Weber has emphasized, it is important in this context to distinguish between *pre-desicursive* and *non-discursive* or *extra-discursive* (*cf.* Weber, 2003, p. 50, note 2): The definition of nature as something pre-discursive has been criticized by postmodern philosophers for repeating naturalistic prejudices and philosophical phantasies of origin (*cf.* Weber, 2003, p. 50; note 2). Contrary to that, reference to nature as non- or extra-discursive is “a theoretical position that assumes that nature is not produced solely by (human) discourse, by culture” (Weber, 2003, p. 50; note 2; my transl.). According to Haraway, we can thus encounter nature both as *topos*, a rhetorical commonplace to which we turn “to *order* our discourse” (PM, p. 65; my emph.), i.e., something that is not itself discursive, and as a *trópos*, a “figure, construction, artifact, movement, displacement,” which is based on a “turn,” so that nature as a *trópos* “cannot pre-exist its construction” (PM, p. 65).

^9^The Actor-Network-Theory has been criticized with regard to various aspects, such as its generous granting of agency to all possible objects (*cf.* Collins and Yearly, 1991; Bloor, 1999; Kneer, 2008) or with regard to the equation of consciousness and intentionality which neglects bodily intentionality (Jonggab, 2020) and more generally, with regard to its indifference to specifically human competence (*cf.* Sayes, 2014, p. 139). For a more neutral overview of the meaning(s) of Actor-Network-Theory’s conception of nonhuman agency, *cf.* Sayes (2014).

^10^However, Haraway criticises Latour for focusing primarily on the interactions of scientists with each other and with their machines and that he consequently “pay[s] too little attention to the non-machine, other non-humans in the interactions” (PM, note 14).

^11^This is of course just a very rough sketch of some approaches in philosophy of agency. For an overview of the more classical views and theories of agency in analytic philosophy, see Schlosser (2019). For a more differentiated overview regarding different forms and problems of agency, including forms of non-human agency, see Ferrero (2022) and therein especially Steward (2022), Thomason and Horty (2022), and van Hateren (2022). For (eco-) feminist accounts of agency outside of knowledge contexts and philosophy of science, see Plumwood (1993, 2001) and Jonggab (2020).

^12^I thank an anonymous reviewer for pointing out the overlaps between Haraway’s and enactivist accounts of agency.

^13^*Cf.* also Lang (2011) offers a detailed discussion of the relation—and a mediation—between traditional and feminist epistemology and applies this to philosophy of education.

^14^Following Haraway, Barad (2007) in her approach called “Agential Realism” has developed a “diffractive methodology” in dialogue with quantum physics. Due to its significance for the wave-particle-duality paradox, she ascribes a key role to the phenomenon of diffraction for understanding “the nature of nature” and also the unstable and entangled subject–object relationship (*cf.* Barad, 2007, esp. Ch. 2). Drawing on Haraway, but above all on Niels Bohr’s philosophy-physics, Barad draws the epistemological consequences of quantum-physical insights with the methodological appropriation of ‘diffraction’ (as opposed to reflection), which consists, among other things, in a dissolution of the subject-object dichotomy and the agential role of scientific apparatuses (*cf.* Barad, 2007, esp. Ch. 3, 4; *cf.* also Ernst, 2016). I am grateful to Waltraud Ernst for drawing my attention to Barad’s work.

^15^The Navajos/Navahos refer to themselves as the Diné, and their ceremonial’s name for the coyote is Áłtsé hashké which means “first scolder” (*cf.* Cooper, 1987, p. 183).

^16^In “Situated Knowledges,” she anticipates some aspects of *Primate Visions* by referring to the reconstructions of the relationship between the body and (female) gender in biology and primatology, in particular *through* “women’s practice as primatologists, evolutionary biologists, and behavioral ecologists” (SK, p. 199): “The *body*, the object of biological discourse, becomes a most engaging being. Claims of biological determinism can never be the same again. When female ‘sex’ has been so thoroughly retheorized and revisualized that it emerges as practically indistinguishable from ‘mind’, something basic has happened to the categories of biology. The biological female peopling current biological behavioral accounts has almost no passive properties left. She is structuring and active in every respect; the ‘body’ is an agent, not a resource” (SK, p. 199 f.).

^17^According to Haraway, one of the dangers in feminist standpoint theory is that of “romanticizing and/or appropriating the vision of the less powerful while claiming to see from their positions” (SK, p. 191). Contrary to that, she thinks that “to see from below is neither easily learned nor unproblematic” (SK, p. 191). The epistemic advantages which standpoint theories grant to oppressed perspectives thus does not result merely *qua* oppression, which means that “[t]he positions of the subjugated are not exempt from critical re-examination, decoding, deconstruction, and interpretation […] [t]he standpoints of the subjugated are not ‘innocent’ positions” (SK, p. 191).

^18^Note that Plumwood does not refer to Haraway’s concept of artifactual nature as fact and fiction here. Even though Plumwood appreciates Haraway’s conception of nature as agent in “Situated Knowledges,” she is critical of Haraway’s later conception of nature in *Modest_Witness@Second_Millenium* (1997; *cf.* Plumwood, 2001, p. 25). Nevertheless, it seems to me that Haraway’s earlier reconception of nature as an active, co-constructed artifactual nature can be illuminated well here with Plumwood’s de−/naturalisation strategies, even though there are important differences between their approaches.

^19^*Cf.* www.umckaloabo.de and www.schwabe.de.

^20^The oncomouse is a laboratory mouse that has been genetically modified in such a way that it tends to develop breast cancer, making it a suitable model for cancer research. The company DuPont had financed the research on the genetic modification at Harvard University and was granted an exclusive license for the patent in the USA in the 1980s, later also in Europe, while the patent application was rejected in Canada (*cf.* M_W, p. 98). It was the first time worldwide that a mammal was patented and thus became a human “invention” and property (*cf.* M_W, p. 79). With the patenting, the animal was marketed as a product—even as a ‘customizable’ product—under the brand name ‘OncoMouse™’ as a trademark. This not only led to protests on ethical grounds from animal rights activists but was also criticized because research with these animals was only possible for institutions that could afford the animal (*cf.* M_W, pp. 96–101).

## References

[ref1] Anscombe G. E. M. (1957). Intention. Cambridge, MA: Harvard University Press.

[ref2] Barad K. (2007). Meeting the universe Halfway. Quantum physics and the entanglement of matter and meaning. Durham: Duke University Press.

[ref3] Bloor D. (1999). Anti-Latour. Stud. History Philos. Sci. Part A 30, 81–112. doi: 10.1016/S0039-3681(98)00038-7, PMID: 11623976

[ref4] Braidotti R. (2018). A theoretical framework for the critical Posthumanities. Theory Cult. Soc. 26, 31–61. doi: 10.1177/0263276418771486

[ref5] Code L. (1995). Rhetorical spaces. Essays on gendered locations. London: Routledge.

[ref6] Collins H. M. Yearly S. (1991). “Epistemological chicken” in A. Pickering science as practice and culture. ed. Pickering A. (Chicago, IL: UCP), 301–326.

[ref7] Cooper G. H. (1987). Coyote in Navajo religion and cosmology. Can. J. Native Stud. VII 2, 181–193.

[ref8] Davidson D. (1963). “Actions, reasons, and causes”, reprinted in Davidson D. (2001). Essays on actions and events (Oxford: Oxford University Press), 3–20.

[ref9] Davidson D. (1971). “Agency” in Agent action, and reason. eds. Brinkley R. Bronaugh R. N. Marras A. (Toronto: University of Toronto Press), 1–37.

[ref10] DeJesus P. (2018). Thinking through enactive agency -sense-making, bio-semiosis and the ontologies of organismic worlds. Phenomenol. Cogn. Sci. 17, 861–887. doi: 10.1007/s11097-018-9562-2

[ref11] DiPaolo E. (2023). F/acts – ways of enactive Worldmaking. J. Conscious. Stud. 30, 159–189. doi: 10.53765/20512201.30.11.159

[ref12] Elgin C. (2017). True enough. Cambridge, MA: MIT Press.

[ref13] Elgin C. (2019). Nominalism, realism and objectivity. Synthese 196, 519–534. doi: 10.1007/s11229-016-1114-0

[ref14] EPO. (2008). Kommune in Südafrika wirft deutscher Firma Bio-Piraterie vor. Entwicklungspolitik. Available at: https://www.epo.de/index.php?option=com_content&view=article&id=3813:kommune-in-suedafrika-wirft-deutscher-firma-bio-piraterie-vor&catid=46&Itemid=115 (Accessed November 23, 2023).

[ref15] Ernst W. (2016). Menschliche und weniger menschliche Verbindungen: Posthumanismus und Gender. FlfF – Kommunikation 33, 37–41. doi: 10.25595/450

[ref16] Ferrero L. (Ed.) (2022). The Routledge handbook of philosophy of agency. London: Routledge.

[ref17] Groenewald Y. (2010). Town like Alice takes on German ‘biopirate’. Mail and Guardian, Available at: https://mg.co.za/article/2010-01-22-town-like-alice-takes-on-german-biopirate/ (Accessed November 23, 2023).

[ref18] Haraway D. (2000). How like a leaf. An interview with Thyrza Nichols Goodeve. New York: Routledge.

[ref19] Haraway D. (2004). “Cyborgs, coyotes, and dogs: a kinship of feminist figurations and there are always more things going on than you thought! Methodologies as thinking technologies. An interview with Donna Haraway conducted in two party”, Lykke NinA Markussen Randi Olesen Finn, in Haraway D., The Haraway Reader (London: Routledge), 321–342.

[ref20] Haraway D. Goodeve T. N. (2018). “Haraway in Conversation with Thyrza Nichols Goodeve” in Modest_Witness@Second_Millenium – FemaleMan meets OncoMouse. ed. Haraway D. (London: Taylor & Francis), xiii–xlviii.

[ref21] Harding S. (1986). The science question in feminism. Ithaca: Cornell University Press.

[ref22] Harding S. (1991). Whose science? Whose knowledge? Thinking from Women’s lives. New York: Cornell University Press.

[ref23] Hoppe K. (2021). Die Kraft der Revision. Epistemologie, Ethik und Politik bei Donna Haraway. Frankfurt a. M: Campus Verlag.

[ref24] Hunter D. (2022). “Epistemic agency” in The Routledge handbook of philosophy of agency. ed. Ferrero L. (London: Routledge), 226–233.

[ref25] Hynes W. J. (1997). “Mapping the characteristics of mythic tricksters: a heuristic guide” in Mythical trickster figures – Contours, contexts, and criticisms. eds. Hynes W. J. Doty W. G. (Tuscaloosa, Alabama: University of Alabama Press), 33–45.

[ref26] Jonggab K. (2020). The problem of nonhuman agency and bodily intentionality in the Anthropocene. Neohelicon 47, 9–16. doi: 10.1007/s11059-020-00534-1

[ref27] Kneer G. (2008). “Hybridizität, zirkulierende Referenz, Amoderne? Eine Kritik an Burno Latours Soziologie der Assoziationen” in Bruno Latours Kollektive. Kontroversen zur Entgrenzung des Sozialen. eds. Kneer G. Shroer M. Schüttpelz E. (Frankfurt: Suhrkamp), 261–305.

[ref28] Lang J. (2011). Epistemology of situated Knoweldges: ‘troubling’ knowledge in philosophy of education. Educ. Theory 61, 75–96. doi: 10.1111/j.1741-5446.2011.00392.x

[ref29] Latour B. (2005). Reassembling the social. An introduction to actor-network-theory. Oxford: Oxford University Press.

[ref30] McAlister F. J. (2010). “Donna J. Haraway (1944-)” in From Agamben to Zizek. Contemporary critical theorists. ed. Simons J. (Edinburgh: Edinburgh University Press), 127–143.

[ref31] Minh Hà T. T. (1986/1987). She, the inappropriate/d other. Discourse 8.

[ref32] Plumwood V. (1993). Feminism and the mastery of nature. London: Routledge.

[ref33] Plumwood V. (2001). Nature as agency and the prospects for a progressive naturalism. CNS 12, 3–32. doi: 10.1080/104557501101245225

[ref34] Prins B. (1997). The standpoint in question – situated knowledges and the Dutch minorities discourse. [Dissertation Thesis]. Available at: www.baukjeprins.nl/publicaties/boeken (Accessed December 2, 2023).

[ref35] Ryle G. (1949). The concept of mind. London: Routledge.

[ref36] Sayes E. (2014). Actor-network theory and methodology: just what does it mean to say that nonhumans have agency? Soc. Stud. Sci. 44, 134–149. doi: 10.1177/0306312713511867, PMID: 28078973

[ref1003] Schlosser M. (2019). “Agency” in Stanford Encyclopedia of Philosophy. Available at: https://plato.stanford.edu/archives/win2019/entries/agency/

[ref37] Singer M. (2005). “Geteilte Wahrheit” in Feministische Epistemologie, Wissenssoziologie und Cultural Studies (Wien: Löcker).

[ref38] Sosa E. (2015). Judgment and agency. Oxford: OUP.

[ref39] Stephen J. (2021). Objectivity in science. Cambridge: CUP.

[ref40] Steward H. (2022). “Animal Agency” in The Routledge handbook of philosophy of agency. ed. Ferrero L. (London: Routledge), 101–108.

[ref41] Taylor P. Maaling S. Coleman S. (2005). The strange story of Umckaloabo. Pharm. J. 275, 790–792.

[ref42] The Human Genome Project (2014). Available at: https://www.genome.gov/human-genome-project (Accessed November 23, 2023).

[ref43] Thomason R. Horty J. (2022). “Artificial and machine agency” in The Routledge handbook of philosophy of agency. ed. Ferrero L. (London: Routledge), 366–375.

[ref44] Trächtler J. (2023). “Speaking in monster tongues: Wittgenstein and Haraway on nature, meaning and the ‘we’ of feminism”, in Feminism: Variants and variations, ed. Lobo C. Silva J. Estevas da, Special issue of Forma de Vida, Available at: https://formadevida.org/jtrachtlerengfdv25 (Accessed December 15, 2023).

[ref45] van Hateren H. (2022). “Minimal agency” in The Routledge handbook of philosophy of agency. ed. Ferrero L. (London: Routledge), 91–100.

[ref46] Weber J. (2003). Umkämpfte Bedeutungen. Naturkonzepte im Zeitalter der Technoscience. Frankfurt: Campus Verlag.

[ref47] Weisberg Z. (2009). The broken promises of monsters. Haraway, animals and the humanist legacy. J. Crit. Anim. Stud., 7, 22–62.

[ref48] Wirth S. (2015). “‘Laborratte’ oder ‘worker’ im Vivisektionslabor? Zur Kontroverse um Donna Haraways Konzeptionen von Agency und ihrer Kritik an Tierrechten” in Das Handeln der Tiere. Tierliche Agency im Fokus der Human-Animal Studies. eds. Wirth S. Laue A. Kurth M. Dornenzweig K. Bossert L. Balgar K. (Berlin: DeGruyter), 115–135.

[ref1002] Wittgenstein L. (1998). On Certainty. eds. Anscombe G. E. M. von Wright G. H. (Oxford: Blackwell).

